# Multiple roles of core protein linker in hepatitis B virus replication

**DOI:** 10.1371/journal.ppat.1007085

**Published:** 2018-05-21

**Authors:** Kuancheng Liu, Laurie Luckenbaugh, Xiaojun Ning, Ji Xi, Jianming Hu

**Affiliations:** 1 Department of Microbiology and Immunology, Penn State University College of Medicine, Hershey, PA, United States of America; 2 College of Life Sciences, Zhejiang Sci-Tech University, Hangzhou, China; University of California, San Diego, UNITED STATES

## Abstract

Hepatitis B virus (HBV) core protein (HBc) contains an N-terminal domain (NTD, assembly domain) and a C-terminal domain (CTD), which are linked by a flexible linker region. HBc plays multiple essential roles in viral replication, including capsid assembly, packaging of the viral pregenomic RNA (pgRNA) into nucleocapsids, viral reverse transcription that converts pgRNA to the genomic DNA, and secretion of DNA-containing (complete) virions or genome-free (empty) virions. The HBc linker is generally assumed to act merely as a spacer between NTD and CTD but some results suggest that the linker may affect NTD assembly. To determine its role in viral replication, we have made a number of deletion and substitution mutants in the linker region, in either the presence or absence of CTD, and tested their abilities to support capsid assembly and viral replication in human cells. Our results indicate that the linker could indeed impede NTD assembly in the absence of CTD, which could be partially relieved by partial linker deletion. In contrast, when CTD was present, the linker deletions or substitutions did not affect capsid assembly. Deletion of the entire linker or its C-terminal part resulted in a partial defect in pgRNA packaging and severely impaired viral DNA synthesis. In contrast, deletion of the N-terminal part of the linker, or substitutions of the linker sequence, had little to no effect on RNA packaging or first-strand DNA synthesis. However, the N-terminal linker deletion and two linker substitution mutants were defective in the production of mature double-stranded viral DNA. Secretion of empty virions was blocked by all the linker deletions and substitutions tested. In particular, a conservative linker substitution that allowed mature viral DNA synthesis and secretion of complete virions severely impaired the secretion of empty virions, thus increasing the ratio of complete to empty virions that were secreted. Together, these results demonstrate that the HBc linker region plays critical and complex roles at multiple stages of HBV replication.

## Author summary

The hepatitis B virus (HBV) is a major human pathogen that infects hundreds of millions of people worldwide and represents a major cause of viral hepatitis, liver cirrhosis, and liver cancer. The HBV capsid protein (HBc) plays multiple roles in the viral life cycle and has emerged recently as a major target for developing antiviral therapies against HBV infection. HBc is divided into three separate regions, an N-terminal domain (NTD) responsible for capsid assembly, a C-terminal domain (CTD) that plays critical roles in the specific packaging of the viral pregenomic RNA (pgRNA) into replication-competent nucleocapsids and the subsequent reverse transcription of the pgRNA into the viral genomic DNA, and a linker region between the NTD and CTD. In contrast to the prevailing assumption that the linker merely serves to connect the NTD and CTD, we have discovered here that it plays a critical role in almost every stage of HBV replication. The linker likely exerted its pleiotropic effects via affecting the NTD and CTD as well as via direct interactions with other viral factors independent of the NTD or CTD. Our results thus not only deepen understanding of HBc structure and functions but also implicate the linker as a potential novel target for antiviral development against HBV infection.

## Introduction

Hepatitis B virus (HBV), a major cause of viral hepatitis, liver cirrhosis, and hepatocellular carcinoma [1], replicates a small (ca. 3.2 kb), partially double-stranded (DS), relaxed circular (RC) DNA via reverse transcription of an RNA intermediate, the pregenomic RNA (pgRNA) [2,3]. Virus assembly begins with the formation of an immature nucleocapsid (NC) incorporating the pgRNA and the viral reverse transcriptase (RT), which then undergoes a process of maturation defined as the conversion of the pgRNA first to a single-stranded (SS) DNA and subsequently to the RC DNA, catalyzed by the RT protein [4]. The RC DNA-containing NC is defined as the mature NC, which can be enveloped by the viral envelope proteins and secreted extracellularly as complete virion.

HBc is a small (183 or 185 amino acids depending on the strains, ca. 21 kd) protein that forms the shell of the NC and also plays a critical role at multiple other stages of HBV replication [2,5,6]. It is composed of three regions, an N-terminal domain (NTD), a C-terminal domain (CTD), and a linker that connects the NTD and CTD. NTD encompasses amino acid residues 1–140 and forms the classical assembly domain, generally thought to be necessary and sufficient for capsid assembly [7–9]. CTD encompasses residues from 150 to the C-terminal end, is highly basic (enriched in R, protamine-like), displays non-specific nucleic acid-binding activity [7,10], and is functionally important in pgRNA packaging and reverse transcription but generally thought to be dispensable for capsid assembly [11–14]. Furthermore, CTD is known to undergo dynamic phosphorylation and dephosphorylation, which regulate HBc functions in pgRNA packaging and reverse transcription [15–22].

**Fig 1:**
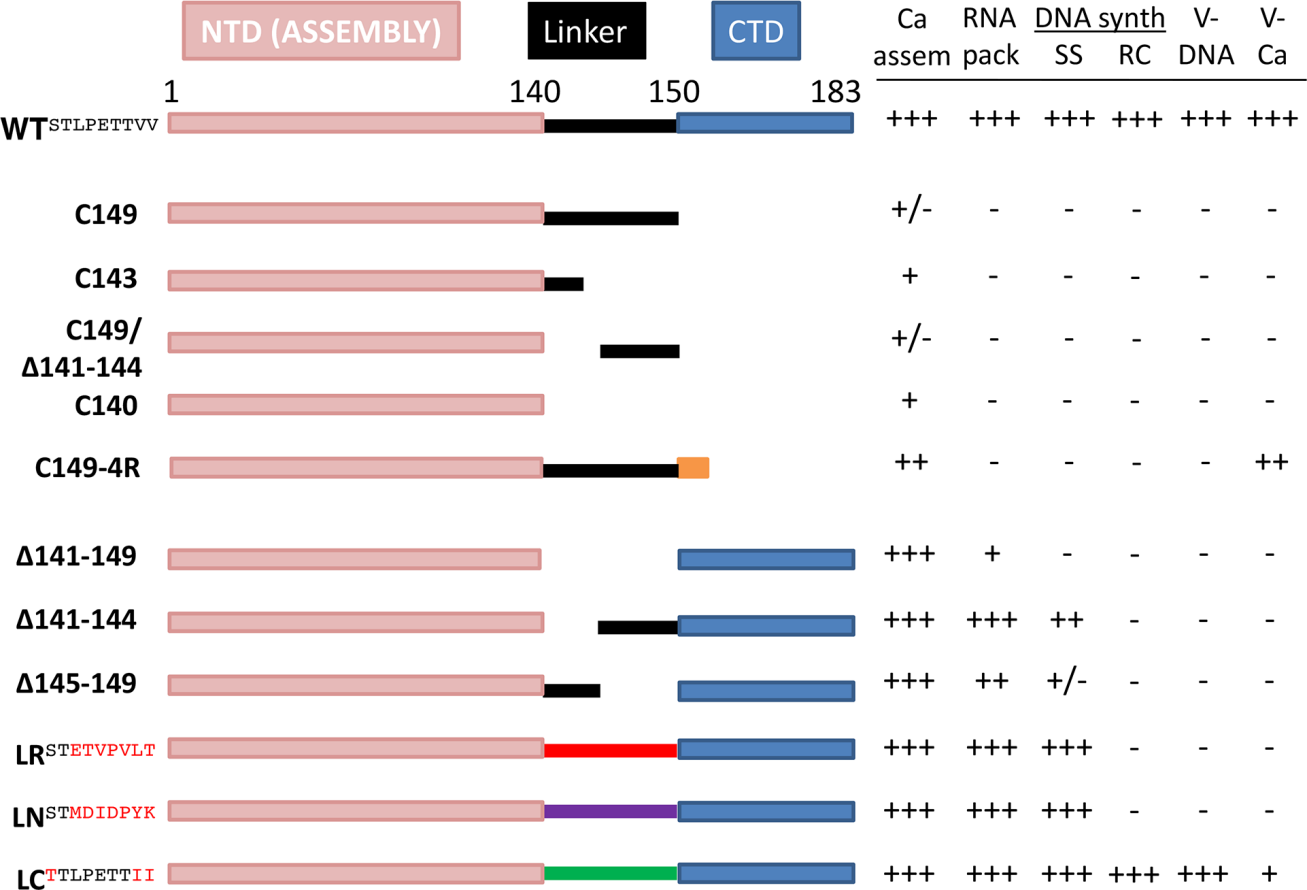
Effects of HBc linker deletions and substitutions on different steps of viral replication. Left. The schematics of the WT HBc (the top line; the linker sequence shown in superscript), and the different deletion and substitution mutants. For the truncations (all from the C-terminal end), the last positions of the mutant proteins are included in the name of the constructs (e.g., HBc149 ending at position 149); for the internal deletions, the residues that are removed are indicated in the names of the mutants (e.g., Δ141–149 removing the entire linker); for the substitutions, the mutant linker sequences are shown in superscript, with the residues different from the WT highlighted in red. The NTD, linker and CTD boundaries are shown on the top. **Right.** The effects of the different HBc mutations on capsid assembly (Ca assem), pgRNA packaging (RNA pack), DNA synthesis (DNA synth), virion DNA (V-DNA; secretion of complete virions), and virion capsid (V-Ca; secretion of empty virions). +++, 50–100% of WT; ++, 10–50% of WT; +, 1–10% of WT; +/-, less than 1% of WT; -, undetectable (by Southern blot analysis for DNA). For the assembly-defective mutants (i.e., those lacking the entire CTD and part or all of the linker), their expression levels were also lower than that of the WT (see text for discussions on possible effects on protein production and/or degradation).

In between the NTD and CTD is a “linker” peptide with a conserved sequence, _141_STLPETTVV_149_ (Fig 1) [23]. The linker is routinely included together with NTD (as in HBc149; Fig 1) for recombinant expression and capsid assembly in bacterial systems and in vitro assembly reactions using HBc proteins purified from bacteria under high protein and/or salt concentration conditions [5]. Under these assembly conditions, the linker clearly does not interfere with NTD assembly. Indeed, deletion of most of the linker (from 143–149) in the context of the full-length HBc, resulting in the fusion of CTD directly to NTD, abolished capsid assembly when expressed in E. coli, suggesting a positive role for the linker in capsid assembly by the full-length HBc [24]. Furthermore, permutation of the last 7 residues of the linker in the context of HBc149 also prevented capsid assembly but replacement of these same seven residues by the seven N-terminal residues of HBc (MDIDPYK) maintained assembly [24]. These results thus further indicated that the specific sequence of the linker can modulate capsid assembly by the full-length HBc under those conditions. On the other hand, the linker can be removed entirely and NTD alone is able to assemble into capsids under those conditions, in the absence of both the CTD and the linker [8,24,25]. Interestingly, truncation of the linker, in the complete absence of the CTD, affected the ratio of the T = 3 (with 90 HBc dimers) or T = 4 (with 120 dimers) capsids assembled under these conditions [24,25]. Thus, whereas most capsids formed when the linker is present belong to the T = 4 class, most capsids formed when the linker is removed belong to the T = 3 class. Other than this apparent effect on the dimorphism of capsid assembly, the mechanism of which remains elusive, the linker is not known to have any other specific functions in HBV replication.

As a para-retrovirus, HBV is selective in virion morphogenesis in that only mature NCs containing the DS, RC DNA, but not immature NCs containing either pgRNA or the SS DNA, are selected for envelopment and secretion as complete virions [26,27]. Situated between the genome and the envelope, the capsid plays an integral role in this selective virion formation process. Within the NTD and spatially located on the surface of the capsid shell, a so-called matrix binding domain (MBD) has been defined, through elegant genetic analysis, that is thought to interact with a short segment in the preS1 region of the viral large envelope protein (L), the so-called matrix domain (MD), for complete virion formation [28–30]. L is one of three HBV surface or envelope proteins (HBs) (the other two being the middle or M and small or S surface protein), which are also secreted as the classical HBsAg particles (the Australian antigen) that contain no capsid or genome, in huge excess over complete virions (by up to 100,000-fold) [27]. Surprisingly, recent studies have revealed that HBV also secretes very high levels (ca. 10^11^/ml) of genome-free (empty) virions, which contain the envelope and capsid but no DNA or RNA and are found at ca. 100-fold excess over complete (i.e., RC DNA-containing) virions in cell culture supernatant and in the blood of experimentally infected chimpanzees and naturally infected humans [27,31,32]. Neither the capsid nor the envelope requirements for empty virion formation are clear at present. Naked (non-enveloped) capsids are also released in cell cultures via an unknown mechanism that appears to be different from that for the secretion of virions [33]. However, the release of naked capsids seems to be a phenomenon in transformed cell lines, and has not been observed in vivo during HBV infection [31,32,34].

We have recently demonstrated that contrary to expectation, the HBc CTD is apparently needed for capsid assembly in living human cells and in the rabbit reticulocyte lysate (RRL), where both the protein concentration and salt conditions mimic more closely the conditions in authentic human host cells than the previous assembly systems using bacterial expression and purified HBc proteins [35]. An HBc construct containing both the NTD and the linker (i.e., HBc149) but no CTD was unable to assemble under these (near) physiological conditions. On the other hand, other CTD-lacking HBc constructs that also lack part of the linker attached to the NTD (truncated at position 147, 145, or 144) accumulated and assembled to varying but detectable levels [11,36–38], indicating that the exact truncation point within the linker region affects the capacity of NTD to assemble in the absence of CTD. These results raise the possibility that the linker sequence can somehow interfere with the assembly by NTD in the absence of CTD under (near) physiological conditions in RRL and in human cells, and this inhibitory effect of the linker on the NTD assembly function is somehow overcome by CTD in the full-length HBc.

Given the unexpected role of CTD, and potentially of the linker, in capsid assembly in RRL and in human cells, it is now important to further assess the role of the CTD, the linker and the interplay between the CTD and the linker, in capsid assembly under physiological conditions. Also, these results bring about the possibility that the linker may have potential roles in the other functions of HBc beyond capsid assembly, which has never been tested so far. Therefore, we have carried out a genetic analysis to test the role of the linker in capsid assembly, both in the presence and absence of CTD, under near physiological conditions in vitro and in cells. Furthermore, the effects of a panel of linker deletion and substitution mutants on pgRNA packaging, DNA synthesis, and virion secretion were assessed. Our results have revealed that the linker indeed can affect capsid assembly in a manner that is dependent on CTD, and furthermore, it plays a critical role in multiple stages of HBV replication beyond capsid assembly.

## Results

### The HBc linker interfered with NTD assembly in human cells in the absence of the CTD

Since previous reports have found that HBc mutants with truncation of the linker region, in addition to CTD removal (i.e., C-terminal truncation beyond 149), could be expressed and assemble at appreciable levels [11,36–38] whereas NTD plus an intact linker (also without CTD) (i.e., HBc149) failed to assemble and accumulate [35,38], we reasoned that deletion of the linker may restore HBc expression and/or assembly, when the CTD was absent. Thus, we deleted the entire linker (from 141 to 149) or only part of the linker (from 144–149), both in the absence of CTD, to make HBc140 and HBc143 (Fig 1), respectively, and determined their expression and assembly in human hepatoma cells (HepG2 and Huh7). A second plasmid expressing the HBV pgRNA and all viral proteins except HBc (HBV-C^-^) was co-transfected in a *trans*-complementation assay to assess the ability of the mutant HBc proteins to carry out the other functions of HBc including pgRNA packaging, DNA synthesis, and virion secretion. We selected the plasmid pSVHBV1.5 to derive the HBc-defective genomic construct, as our pilot experiments showed that this plasmid secreted significantly higher levels of HBsAg than another genomic construct pCMVHBV (S1 Fig). Since the secreted HBsAg is known to be in great excess over virions during natural HBV infection [27,31], the higher levels of HBsAg produced from pSVHBV1.5 helped to ensure that the complementation experiment mimicked better the natural infection in terms of HBsAg expression and to avoid the potential situation where the expression of the envelope proteins might become limiting for virion secretion. Even though the HBc sequence used here was from HBV genotype D, and the complementing (HBV-C^-^) construct was from HBV genotype A, they complemented each other efficiently in all aspects of viral replication assayed here, as shown below.

**Fig 2:**
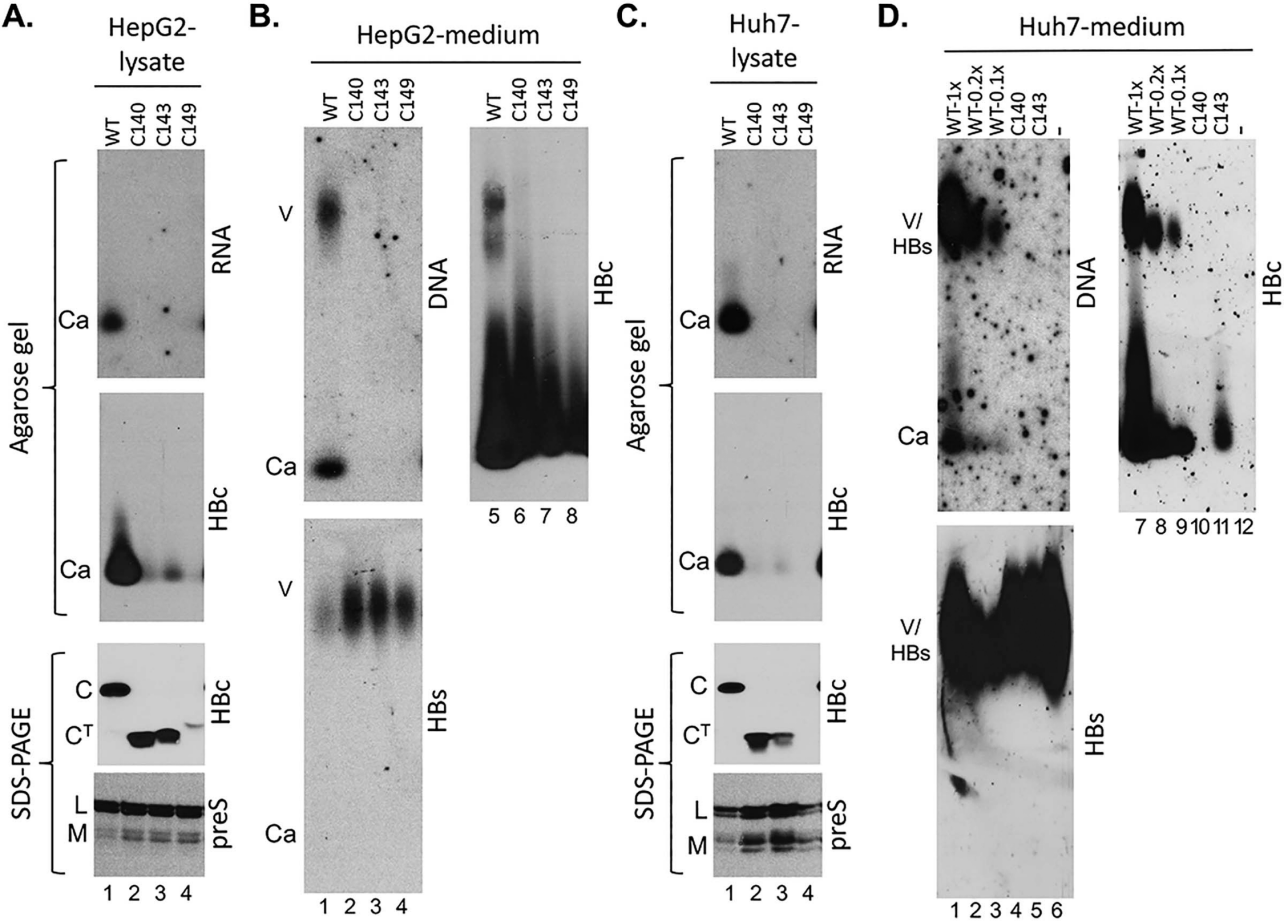
Analysis of HBV capsid assembly and virion secretion by HBc linker deletion mutants in the absence of the CTD. HepG2 (A and B) or Huh7 cells (C and D) were transfected with the indicated HBc expression constructs, together with an HBV genomic construct defective in HBc expression. Seven days later, the culture supernatant as well as the cells were collected. **A and C.** Cytoplasmic lysate was analyzed for capsid assembly, following agarose gel electrophoresis and transfer to nitrocellulose membrane, using the rabbit polyclonal anti-HBc antibody (2^nd^ panel) and pgRNA packaging using the anti-sense HBV RNA probe (top). Total HBc levels were measured, following SDS-PAGE and transfer to PVDF membrane, using the mouse mAb T2221 (3^rd^ panel). Levels of the HBV L and M envelope proteins were detected by the preS2-specific mAb to monitor both transfection efficiency and sample loading (bottom). **B and D**. Concentrated culture supernatant (medium) was analyzed for virion secretion by agarose gel electrophoresis. Following transfer to nitrocellulose membrane, HBV DNA in virions (and naked capsids) released into the medium was detected by using the HBV DNA probe (B and D, top left) and capsid protein by western blot analysis using the rabbit polyclonal anti-HBc antibody (B and D, top right). HBV envelope proteins were detected by using the rabbit anti-HBs antibody (B and D, bottom). In panel D, decreasing amount of the supernatant from the WT HBc transfection was loaded (lanes 1–3, 7–9), with the amount of loading in lanes 1 and 7 equal to that loaded from the mutant HBc140 (C140, lanes 4, 10) and HBc143 (C143, lanes 5, 11) transfection and that loaded in lanes 2, 3, 8, and 9 being 5-fold less and 10-fold less than that loaded in lanes 1 and 7, respectively. The sample loaded in lanes 6 and 12 was from cells transfected only with the HBc-defective genomic construct without the HBc-expressing construct. V, virion; Ca, capsid; C, HBc; C^T^, truncated HBc (HBc140, HBc143, HBc149).

**Fig 3:**
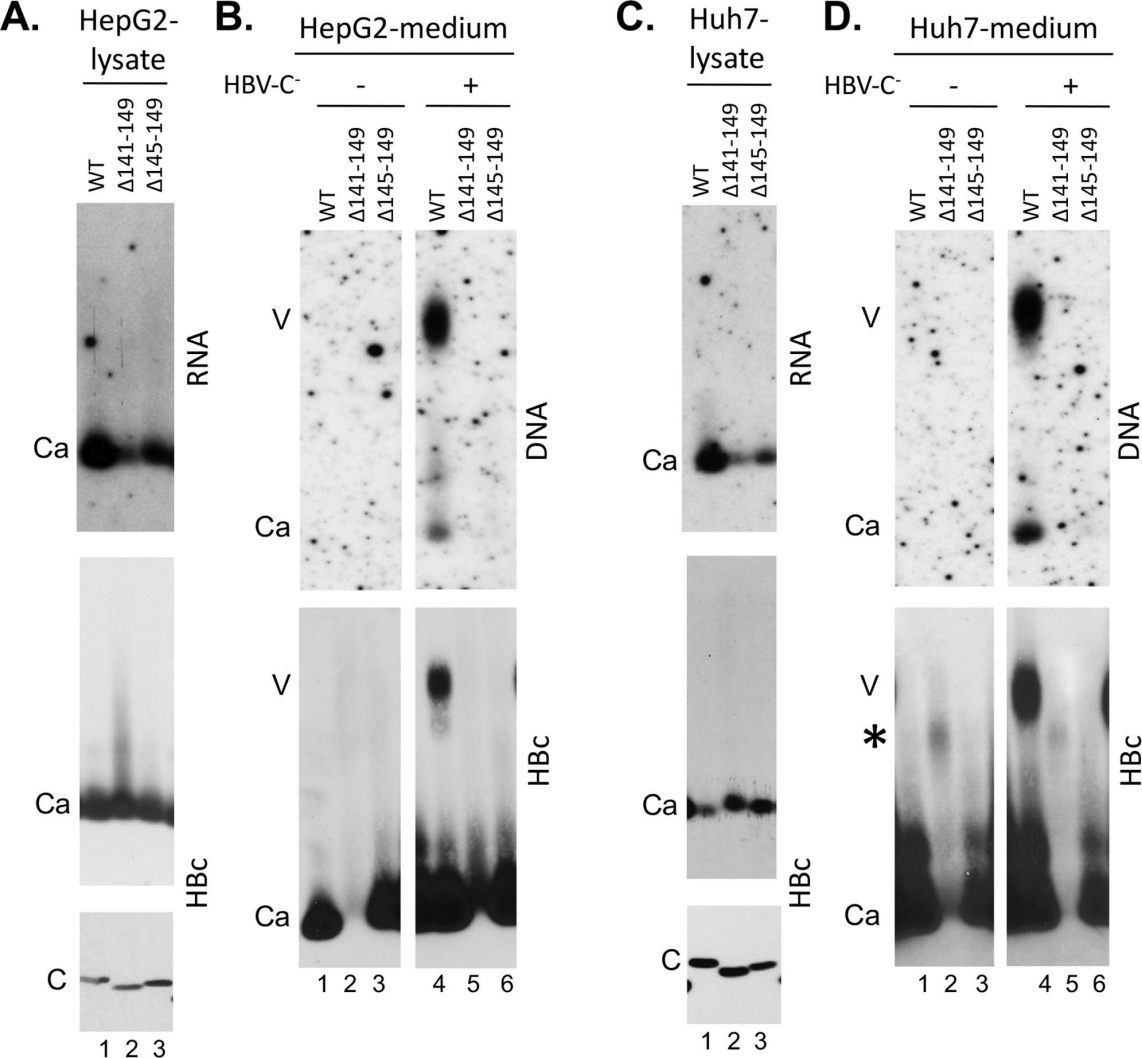
Analysis of HBV capsid assembly and virion secretion by HBc linker deletion mutants in the presence of the CTD. HepG2 (A and B) or Huh7 cells (C and D) were transfected with the indicated HBc expression constructs, either alone (B and D, lanes 1–3) or together with an HBV genomic construct defective in HBc expression (A and C, all lanes; B and D, lanes 4–6). Seven days later, the culture supernatant as well as the cells were collected. **A and C.** Cytoplasmic lysate was analyzed, following agarose gel electrophoresis and transfer to nitrocellulose membrane, for capsid assembly using the rabbit polyclonal anti-HBc antibody (middle) and pgRNA packaging using the anti-sense HBV RNA probe (top). Total HBc levels were measured, following SDS-PAGE and transfer to PVDF membrane, using the mouse mAb T2221 (bottom). **B and D**. Concentrated culture supernatant (medium) was analyzed for virion secretion by agarose gel electrophoresis. Following transfer to nitrocellulose membrane, HBV DNA in virions (and naked capsids) released into the medium was detected by using the HBV DNA probe (top) and capsid protein by using the rabbit polyclonal anti-HBc antibody (bottom). V, virion; Ca, capsid; C, HBc. *, HBc signal migrating slower than naked capsids in supernatant from Huh7 cells transfected with HBc/Δ141–149 (**D**).

In support of a negative effect of the linker on NTD expression/assembly as hypothesized above, the expression levels of HBc140 and HBc143, as assessed by SDS-PAGE and western blot analysis, approached those of the WT HBc in both HepG2 and Huh7 cells (Fig 2A and 2C, 3^rd^ and bottom panels), much better than that of HBc149, which retains the entire linker [35,38]. As shown in Figs 2 and S2, the mAb T2221, recognizing an epitope towards the end of the HBc NTD [39], detected the WT and CTD- (and linker-) deleted HBc proteins very well, in comparison with two other mAbs targeted to the beginning of NTD, 10E11 [40] (commercially available) and the anti-WHc made against the very N-terminal sequences of the woodchuck hepatitis virus (WHV) core protein (WHc), which are identical to those in HBc [32,41] (see Materials and Methods). The levels of intracellular capsids (Fig 2A and 2C, 2^nd^ panels), and the naked capsids released into culture medium (Fig 2B, top right), as assessed by native agarose gel electrophoresis and western blot analysis, were also higher than those of HBc149 although still lower than those of the WT HBc. For HepG2 cells, the naked capsids released into the culture supernatant by HBc140 and HBc143 (and even HBc149) were relatively abundant (though still less than the WT HBc) (Fig 2B, top right) although the levels of intracellular capsids from these mutants were very low (Fig 2A, 2^nd^ panel). Thus, the release of naked capsids into the culture supernatant might be enhanced by the linker (and CTD) deletions in HBc140 and HBc143. An enhanced release of capsids plus a partial defect in capsid assembly could explain the relative abundance of HBc140 and HBc143 proteins detected by SDS-PAGE western blot analysis (Fig 2A, 3^rd^ panel) but very low levels of intracellular capsids (Fig 2A, 2^nd^ panel). For Huh7 cells, a similar phenomenon could have occurred but the released naked capsids from HBc140 (and to a lesser degree, HBc143) could have been rapidly disrupted/degraded in the supernatant (see also Fig 3 below). This could explain the relative abundance of these mutant proteins detected by the SDS-PAGE western blot analysis (Fig 2C, 3^rd^ panel) but very low levels of intracellular and extracellular capsids (esp. for HBc140) (Fig 2C, 2^nd^ panel; Fig 2D, top right).

### The HBc linker was not required for capsid assembly in human cells when both NTD and CTD were present

**Fig 4:**
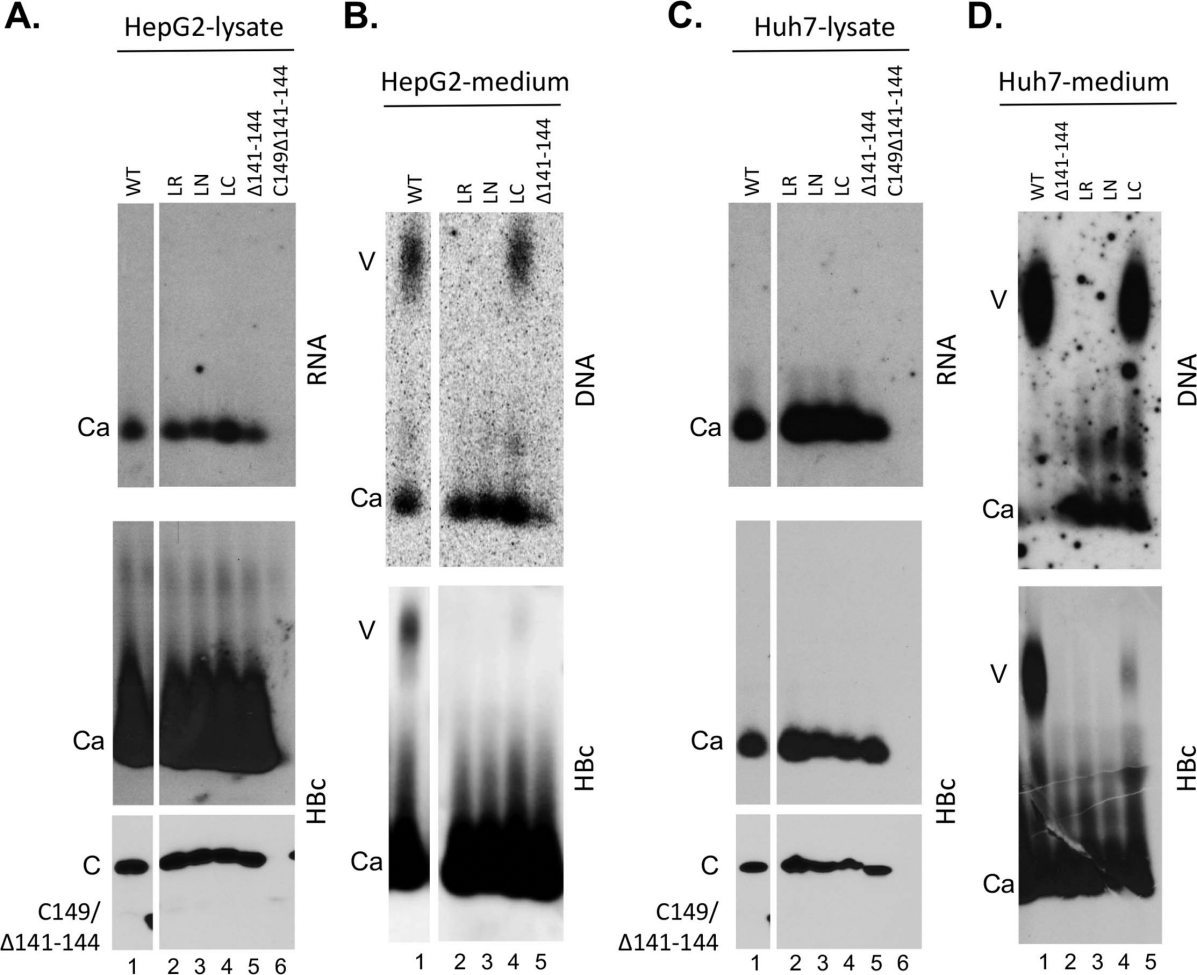
Analysis of HBV capsid assembly and virion secretion by HBc linker substitution and deletion mutants. HepG2 (A and B) or Huh7 cells (C and D) were transfected with the indicated HBc expression constructs, together with an HBV genomic construct defective in HBc expression. Seven days later, the culture supernatant as well as the cells were collected. **A and C.** Cytoplasmic lysate was analyzed, following agarose gel electrophoresis and transfer to nitrocellulose membrane, for capsid assembly using the rabbit polyclonal anti-HBc antibody (middle) and pgRNA packaging using the anti-sense HBV RNA probe (top). Total HBc levels were measured, following SDS-PAGE and transfer to PVDF membrane, using the mouse mAb T2221 (bottom). **B and D**. Concentrated culture supernatant (medium) was analyzed for virion secretion. Following agarose gel electrophoresis and transfer to nitrocellulose membrane, HBV DNA in virions (and naked capsids) released into the medium was detected by using the HBV DNA probe (top) and capsid protein by using the rabbit polyclonal anti-HBc antibody (bottom). V, virion; Ca, capsid; C, HBc.

To assess the potential role of the linker in the context of the full-length HBc (i.e., with both NTD and CTD), we constructed HBc/Δ141–149 (with the entire linker deleted) and HBc/Δ145–149 (deleting the C-terminal portion of the linker), which share the similar linker deletions as HBc140 and HBc143 but retain the CTD (Fig 1). Both of these linker deletion constructs were expressed and assembled into capsids like the WT HBc in both HepG2 and Huh7 cells (Fig 3A and 3C, middle and bottom panels). These results were rather surprising in light of the previous report showing that deletion of the linker, thus fusing the CTD directly to NTD, abolished capsid assembly in the bacterial expression system, which was taken as evidence to indicate a need for a flexible linker between the NTD and CTD to prevent the CTD from interfering with NTD assembly [24]. In light of this surprising result, we constructed another partial linker deletion construct, in the context of the full-length HBc, by deleting HBc residues 141–144 (i.e., the N-terminal portion of the linker) to make HBc/Δ141–144 (Fig 1). In addition, we made the same partial N-terminal deletion of the linker, in the absence of CTD, to construct HBc149/Δ141–144 (Fig 1). HBc/Δ141–144 was expressed and assembled just like the WT HBc in both HepG2 and Huh7 cells (Fig 4A and 4C, lane 5, middle and bottom panels). On the other hand, the same partial linker deletion, in the absence of the CTD, in HBc149/Δ141–144 did not rescue NTD expression or assembly (Fig 4A and 4C, lane 6, middle and bottom panels), unlike the deletion of the entire linker (in HBc140) or its C-terminal portion (in HBc143) described above.

It was previously shown that the sequence of the linker between the NTD and CTD could affect capsid assembly in the bacterial expression system [24]. We thus tested two different linker substitution mutations that were shown to be either compatible or not with assembly (Fig 1). The substitution that disrupted assembly was a randomized WT linker sequence (STETVPVLT, dubbed LR for “linker random” here), whereas the substitution that retained assembly was the replacement of the last seven residues of the linker by the first seven residues from the N-terminal end of HBc with the first two residues unchanged (STMDIDPYK, dubbed LN for “linker N-terminal” here). Interestingly, we found both of these linker substitutions were similar to the WT HBc in expression and assembly in human hepatoma cells (Fig 4A and 4C, lanes 2–3, middle and bottom panels), in contrast to their severe defect in assembly in bacteria [24], further attesting to the drastic effects of the expression host on the assembly behavior of the different HBc constructs. We also made a third linker substitution with a nine-residue segment (TTLPETTII) from a cellular protein (dubbed LC for “linker cellular” here) that is very similar to the WT HBc in sequence (the middle six residues being the same as the WT linker and the other three residues representing conserved substitution: S141T, V148I, and V149I) and in predicted secondary structure [24]. This substitution was also compatible with capsid assembly in hepatoma cells (Fig 4A and 4C, lane 4, middle and bottom panels).

### The HBc linker played an important role, in a sequence-independent manner, in pgRNA packaging

We next tested the potential effect of the linker deletions and substitutions on the HBc function in pgRNA packaging into NCs. Given the known critical role of CTD in mediating pgRNA packaging, it was no surprise that none of the CTD deletion mutants with or without linker deletions (HBc140, HBc143, HBc149, HBc149/Δ141–144) were able to support packaging of viral RNA (Fig 2A and 2C, lanes 2–4, top panels; Fig 4A and 4C, lane 6, top panels). On the other hand, it was interesting that some of the linker mutations, in the presence of an intact CTD, also impaired pgRNA packaging. The complete linker deletion, HBc/Δ141–149, showed a decrease in pgRNA packaging by ca. 5–10 fold after normalizing to the amount of capsids (Fig 3A and 3C, lane 2, top panels), whereas partial deletion of the C-terminal portion of the linker, HBc/Δ145–149, decreased pgRNA packaging less severely, by ca. 3–4 fold (Fig 3A and 3C, lane 3, top panels). Partial deletion of the N-terminal portion of the linker, HBc/Δ141–144 had the weakest effect, decreasing pgRNA packaging by ca. 2 fold (Fig 4A and 4C, lane 5, top panels). In contrast to the linker deletions, none of the linker substitutions affected pgRNA packaging (Fig 4A and 4C, lanes 2–4, top panels), indicating that the specific sequence of the linker was not critical for this HBc function.

### The HBc linker played an important role, in a sequence-independent and -dependent manner, at different stages of viral DNA synthesis

As expected from the essential role of CTD in pgRNA packaging as well as in facilitating viral reverse transcription, none of the CTD deletion mutants (HBc140, HBc143, HBc149, HBc149/Δ141–144) showed any viral DNA in NCs (Figs 1, 2B and 2D, top left, lanes 2–4). Intriguingly, even in the presence of the CTD, the complete linker deletion (HBc/Δ141–149) and the C-terminal partial linker deletion (HBc/Δ145–149) showed no viral DNA in NCs (Fig 3B and 3D, lanes 5, 6, top panels), indicating a critical role of the linker, particularly its C-terminal portion (145–149), in viral reverse transcription beyond its role in facilitating pgRNA packaging described above. On the other hand, the N-terminal partial linker deletion, HBc/Δ141–144, contained some viral DNA in NCs, although at reduced levels compared to the WT HBc (Figs 1 and Fig 4B, lane 5, top panel). The three linker substitutions apparently contained viral DNA in their capsids at levels similar to the WT (Fig 4B, lanes 2–4, top panel; Fig 4D, lanes 3–5, top panel).

**Fig 5:**
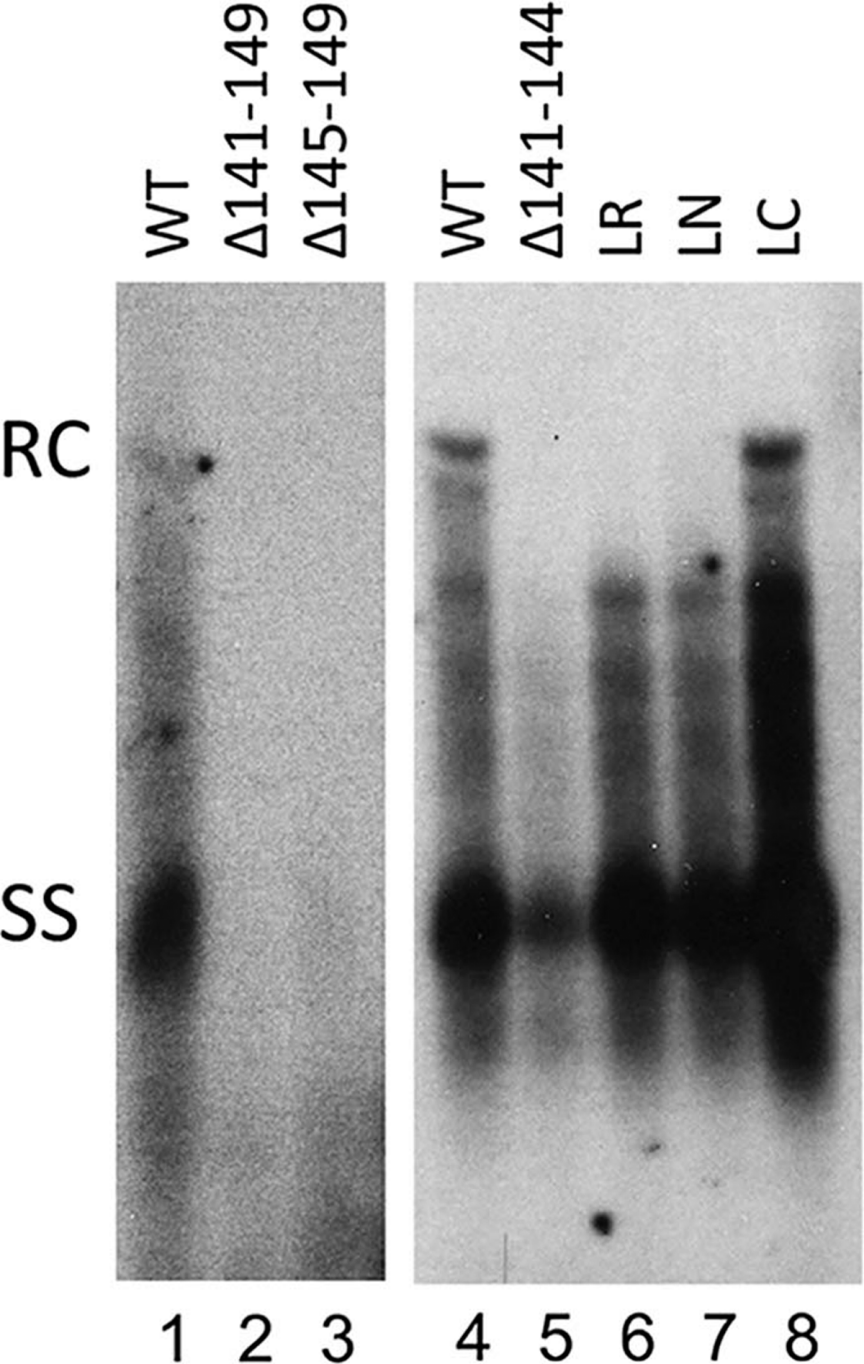
Analysis of HBV DNA synthesized by HBc linker deletion and substitution mutants. HepG2 cells were transfected with the indicated HBc expression constructs, together with an HBV genomic construct defective in HBc expression. Seven days later, the transfected cells were harvested. HBV NC-associated DNA (core DNA) was extracted from cytoplasmic lysate (without nuclease digestion, See Materials and Methods for details) and detected by Southern blot analysis. Input plasmid DNA (but not viral replicative DNA) was removed with DpnI digestion before Southern blot analysis. RC, RC DNA,; SS, SS DNA.

To assess the species of DNA synthesized in mutant capsids, we extracted viral DNA from the WT and mutant capsids and analyzed their DNA content by Southern blot analysis. Previous results from us and others indicated that certain capsid mutants allow viral DNA synthesis but are unable to protect their DNA content from exogenous nuclease digestion, which is routinely used to remove plasmid DNA during core DNA extraction [19,42,43]. To avoid this potential issue so as to obtain a more accurate assessment of viral DNA synthesized in the mutant capsids, we extracted capsid-associated DNA (or core DNA) without nuclease digestion but then degraded the contaminating plasmid DNA in the resulting core DNA preparation with DpnI, which digests plasmid DNA (methylated in bacteria) but not viral DNA synthesized in hepatoma cells [42]. All capsids that contained viral DNA (the three linker substitutions and the partial N-terminal linker deletion, HBc/Δ141–144) based on the particle gel analysis (Fig 4B and 4D) had SS DNA (i.e., minus strand), although the SS DNA levels were reduced in HBc/Δ141–144 by ca. 2-fold compared to the WT HBc (Fig 5). As the SS DNA is reverse transcribed from pgRNA, this modest reduction of SS DNA in HBc/Δ141–144 was at least partly due to the moderately reduced levels of pgRNA packaging in this mutant described above. In contrast, HBc/Δ141–149 showed no DNA and HBc/Δ145–149 showed barely detectable levels SS DNA (Fig 5), consistent with the particle gel results (Fig 3B and 3D). Again, this DNA synthesis defect could be partly the consequence of the defect in pgRNA packaging by these two mutants. These results thus indicated that the specific sequence of the linker was not critical for the first step of reverse transcription to generate the minus strand DNA, and a linker that was only five (instead of the nine in WT) residues long was sufficient for SS DNA synthesis. Intriguingly, the partial N-terminal linker deletion (HBc/Δ141–144), as well as two linker substitutions (LR and LN), showed no RC DNA in their capsids in contrast to the WT HBc (Fig 5, lanes 5–7). These three mutants did make immature DS DNA intermediates (running as a smear between the SS DNA and RC DNA in Fig 5), indicating they were able to initiate plus strand DNA synthesis and elongate the plus strand to a limited extent. However, only the conservative linker substitution (LC) was competent in RC DNA synthesis (Fig 5, lane 8), thus implicating a critical role of the linker, in a sequence-specific manner, in the second step of reverse transcription (extensive plus strand DNA synthesis to generate RC DNA).

### The HBc linker played a critical role, in a sequence-dependent manner, in virion secretion

We next assessed the capacity of the linker mutants to be enveloped and secreted into the culture supernatant as virions. Viral particles (including both virions and naked capsids) released into the culture supernatant of transfected HepG2 or Huh7 cells were analyzed by native agarose gel electrophoresis, whereby naked (non-enveloped) capsids released into the culture supernatant were well separated from virions (enveloped) as the former migrated much faster than the latter on the gel (Figs 2–4, panels B and D). Complete virions were detected by Southern blot analysis of HBV DNA. Empty virions were detected by western blot analysis of the HBc protein in virions, assuming that the vast majority of HBc signal (99% or more) from virions was from empty virions, as shown in previous studies [18,31,32]. As expected, HBV DNA in (complete) virions (or naked capsids), readily detectable in WT virions, was not detected from HBc140, HBc143 or HBc149 in HepG2 cells (Fig 2B, top left) (true also for Huh7 cells; see below Fig 2D, top left), due to their lack of CTD, which is known to be essential for pgRNA packaging or DNA synthesis. On the other hand, the HBc protein signal detected in the WT virions (i.e., empty virions) was also undetectable from these mutants when tested in HepG2 cells (Fig 2B, top right). These results thus indicated that the linker, and/or CTD (see below also), was important for secretion of empty virions.

This suggestion was then confirmed by results obtained using Huh7 cells, when decreasing amounts of culture supernatant from the WT HBc transfection were analyzed along with that from the HBc140 and HBc143 transfection (Fig 2D). When the amount of supernatant from the WT HBc transfection was decreased by 10-fold, the levels of naked capsids released into the medium were similar to those from the HBc143 transfection (Fig 2D, top right, lanes 3 and 5); virion capsids were clearly detectable from the WT HBc even with this reduced loading whereas no virion capsids from either HBc143 or HBc140 were detected (Fig 2D, top right, lanes 3–5). As expected, the HBs signals were only detected with virions but not naked capsids (Fig 2B and 2D, bottom). As HBsAg particles (with no capsids or genome) are not separated from virions (either empty or complete) on the agarose gels under these conditions [31,32], the abundant HBsAg signals, in the absence of HBc signals at the top of the gel in the case of HBc140, HBc143 and HBc149 represented just HBsAg particles (no virions) (Fig 2B, lanes 2–4, bottom; Fig 2D, lanes 4, 5, bottom), as verified by the detection of HBsAg at the same position on the gel in the complete absence of HBc expression (Fig 2D, lane 6, bottom). These results thus indicated that capsids formed by NTD, in the absence of CTD and the linker, could not be enveloped for secretion as empty virions.

It was noticeable that the complete linker deletion (HBc/Δ141–149) showed little to no naked capsids in the culture supernatant either (Fig 3B and 3D, lanes 2 and 5), suggesting that the complete linker deletion might also have blocked the release of naked capsids into the culture medium, or if released, was rapidly degraded in the supernatant. On the other hand, we detected a smeary HBc signal migrating just below the virions and much slower than naked capsids, detectable only from this mutant (in Huh7 but not HepG2 cells), in a manner that was independent of the viral envelope proteins (Fig 3B and 3D, lanes 2 and 5, bottom). This result suggested that some naked mutant capsid might be disrupted once released extracellularly under certain conditions. The exact nature of the slowly-migrating HBc smear (in a non-capsid form) from this mutant, and its apparent cell line dependence, remained unclear. If the HBc/Δ141–149 capsid was indeed blocked from release from the cell, the excess mutant capsid might be degraded intracellularly such that its level in the cell did not exceed that of the WT HBc (Fig 3A and 3C).

The role of the linker in virion secretion, both complete and empty, was assessed in the context of HBc linker mutants which retain an intact CTD. Both the complete linker deletion (HBc/Δ141–149) and the two partial linker deletions (HBc/Δ141–144 and HBc/Δ145–149), despite being competent for capsid assembly intracellularly, did not show any virion secretion (Fig 3B and 3D, lanes 5, 6; Fig 4B, lane 5; Fig 4D, lane 2). As two of these three mutants (HBc/Δ141–149 and HBc/Δ145–149) failed to synthesize any viral DNA and the third linker deletion mutant (HBc/Δ141–144) failed to make RC DNA (which is a prerequisite for complete virion secretion) (Figs 3–5), the specific effect of these mutants on secretion of complete virions could not be ascertained from these experiments. However, these results clearly indicated that both parts of the linker were required for secretion of empty virions. The critical role of the linker in virion secretion was further confirmed with the linker substitution mutants. All three linker substitution mutants were defective in secreting empty virions (Fig 4B, lanes 2–4, bottom; Fig 4D, lanes 3–5, bottom), although the conservative substitution (LC) showed a low level of empty virions (ca. 10% of WT) (Fig 4B, lane 4, bottom; Fig 4D, lane 5, bottom). Again, since the LR and LN substitution mutants failed to make RC DNA (Fig 5), the specific effects of these mutations on DNA virion secretion could not be determined from these experiments. Interestingly, the conservative substitution (LC) allowed secretion of complete virions (virion DNA), despite severely blocking the secretion of empty virions (virion HBc) (Fig 4B, lane 4; Fig 4D, lane 5).

### The HBc linker could support empty virion secretion in the absence of a complete CTD

**Fig 6:**
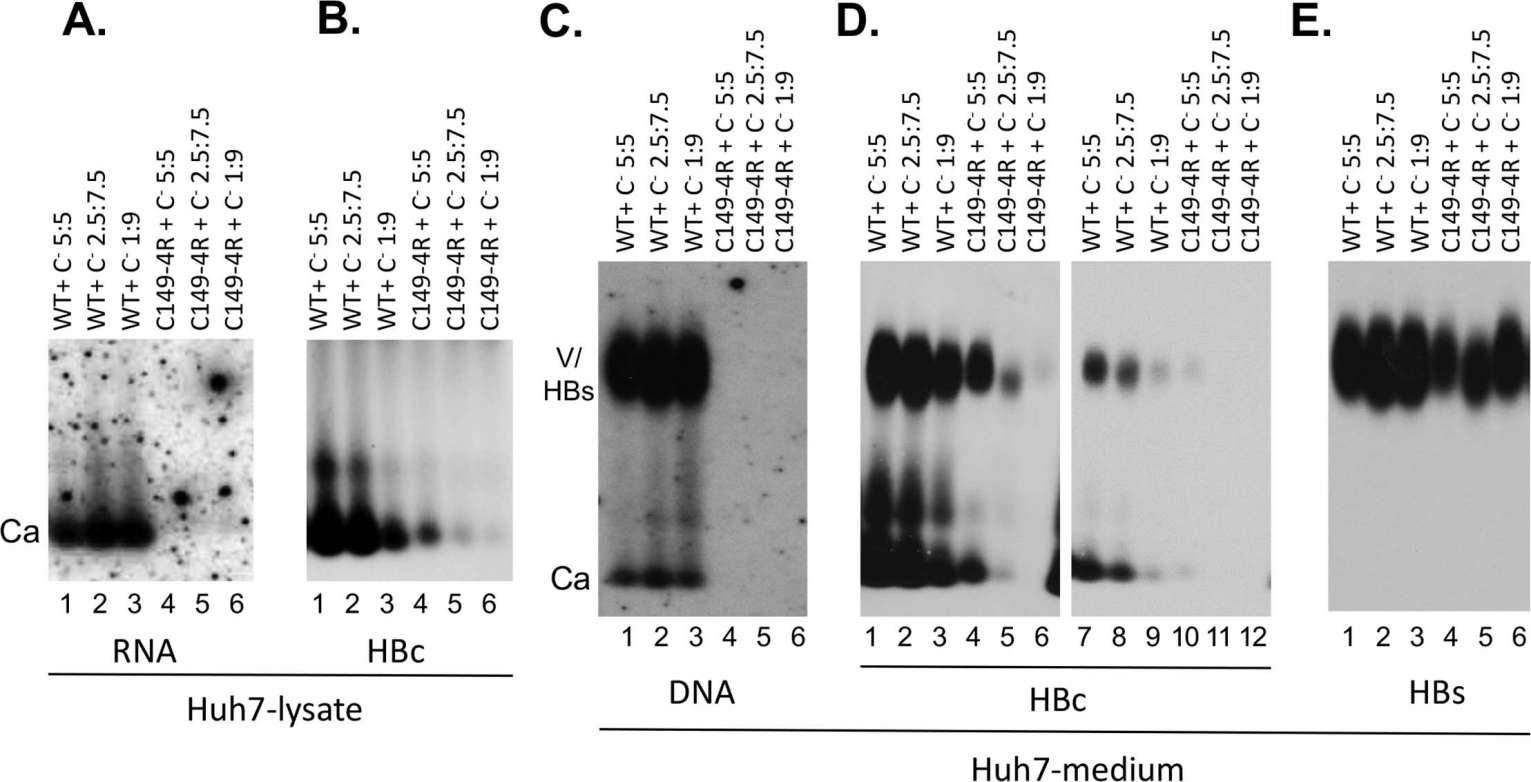
Analysis of capsid assembly and virion secretion by HBc149-4R. Huh7 cells were transfected with an expression construct for the WT HBc or HBc149-4R, together with an HBV genomic construct defective in HBc expression (C^-^) at the indicated ratios (in µg amounts; total amount of DNA per dish kept at 10 µg). Seven days later, the culture supernatant as well as the cells were collected. **A and B.** Cytoplasmic lysate was analyzed for capsid assembly, following agarose gel electrophoresis and transfer to nitrocellulose membrane, using the rabbit polyclonal anti-HBc antibody (**B**) and pgRNA packaging using the anti-sense HBV RNA probe (**A**). **C-E**. Concentrated culture supernatant (medium) was analyzed for virion secretion. Following agarose gel electrophoresis and transfer to nitrocellulose membrane, HBV DNA in virions and naked capsids released into the medium was detected by using the HBV DNA probe (**C**), the capsid protein by using the rabbit polyclonal anti-HBc antibody (**D**), and the envelope proteins by the anti-HBs antibody (**E**). Lanes 7–12 represent a shorter exposure of lanes 1–6 in **D**. V, virion; Ca, capsid.

The results presented above indicated that the linker was required for virion secretion (Figs 3B and 3D and 4B and 4D), but a role for CTD could not be excluded, since when the CTD alone was deleted and the linker was retained (as in HBc149), there was little to no accumulation of intracellular capsids (Fig 2A and 2C, 2^nd^ panel), precluding an assessment of its virion secretion capacity in the absence of the CTD. To overcome this limitation, we appended four positive R residues (4R) to HBc149, reasoning that the supply of the positive charges might rescue assembly of HBc149, in the absence of CTD, by either interacting with non-specific RNA or with NTD of HBc [35,44]. Indeed, HBc149-4R, in contrast to HBc149, accumulated substantial, though still lower than WT, levels of intracellular capsids that were released in the culture medium (Fig 6B and 6D). As expected, the HBc149-4R mutant capsids failed to package pgRNA or synthesize viral DNA due to the lack of a complete CTD (Fig 6A and 6C). Since the capsid levels of HBc149-4R were still lower than those of the WT HBc, we titrated the amount of WT HBc and HBc149-4R plasmids used for transfection, relative to the HBc-defective genomic construct, and measured levels of capsids and virions across the titration to facilitate a direct comparison of virion secretion efficiency of HBc149-4R relative to the WT HBc. Importantly, HBc149-4R, was secreted as virions (empty) as efficiently as the WT, when normalized to the capsid levels (Fig 6B and 6D). Thus, the linker was able to support efficient secretion of (empty) virions in the absence of a complete CTD.

### The HBc linker could modulate CTD phosphorylation

Since the state of CTD phosphorylation is known to play a critical role in capsid assembly, pgRNA packaging, and reverse transcription, which were affected by the linker mutants studied here, we decided to test if the various linker mutants could affect the CTD phosphorylation state. Since HBc assembles into capsid particles rapidly in hepatoma cells, which can affect CTD phosphorylation state indirectly by influencing the accessibility of the CTD phosphorylation sites to host kinases and phosphatases, and CTD also undergoes dynamic phosphorylation and dephosphorylation associated with pgRNA packaging and reverse transcription, we decided to use the RRL in vitro translation system for HBc expression and assembly that we developed recently [35]. In this cell-free system, HBc is phosphorylated during translation by endogenous cellular kinases, at (at least) some of the same CTD sites as in vivo, which is independent of capsid assembly, pgRNA packaging or DNA synthesis [35], and HBc assembly does not occur until triggered by exogenous phosphatase treatment. We therefore examined the CTD state of phosphorylation of the WT HBc and various linker mutants immediately after translation, before triggering capsid assembly, to determine HBc phosphorylation state in the absence of capsid assembly.

**Fig 7:**
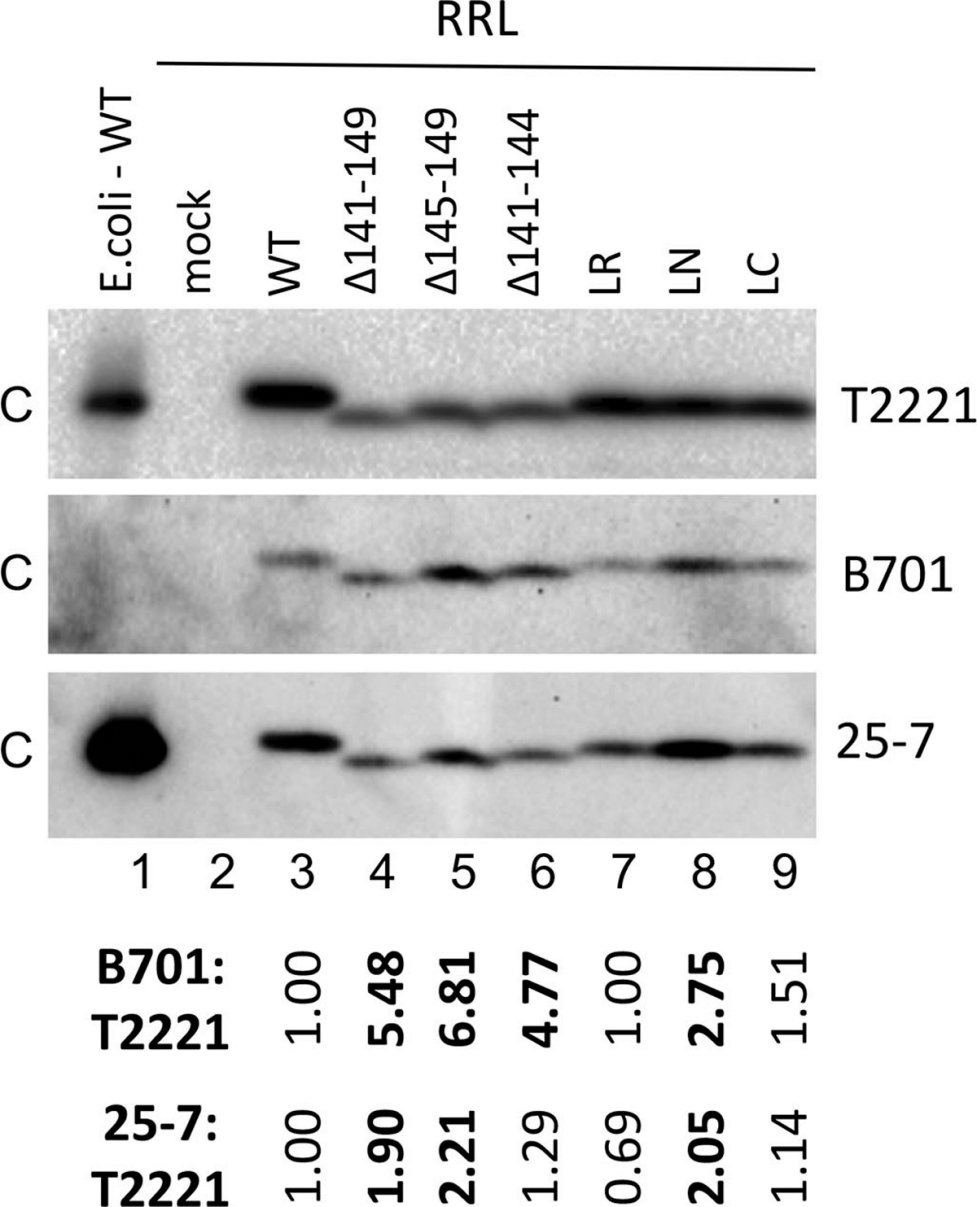
Effects of HBc linker mutants on CTD state of phosphorylation in the RRL in vitro translation system. The WT HBc, or the linker deletion and substitution mutants were translated in RRL. The translated WT and mutant HBc proteins (lanes 3–9), together with the mock translation (lane 2; no template DNA added during translation) and the HBc protein purified from E. coli (lane 1), were resolved by SDS-PAGE and then detected by western blot analysis, using the NTD-specific mAb T2221 (top panel), and two CTD-specific mAbs, B701 (middle panel) and 25–7 (bottom panel). The B701 signal and 25–7 signal were quantified and normalized to the corresponding T2221 signal (B701:T2221 or 25–7:T2221). The normalized B701 or 25–7 signals of the deletion or substitution HBc mutants relative to that of the WT HBc, which was set to 1.00, are listed at the bottom. C, HBc.

Following resolution of HBc by SDS-PAGE, we used an NTD-specific mAb (T2221) to measure the total HBc levels, irrespective of CTD state phosphorylation (Fig 7, top panel) and two CTD-specific mAbs, B701 that is selective for the phosphorylated CTD with an epitope between 155–164 (Fig 7, middle panel), and 25–7 that is selective for the non-phosphorylated CTD with an epitope between 164–182 (Fig 7, bottom panel) [18,35], for western blot analysis. The specificity of the mAbs was verified by using the non-phosphorylated HBc protein purified from E. coli (Fig 7, lane 1). The complete linker deletion mutant (HBc/Δ141–149), as well as the two partial deletion mutants (HBc/Δ141–144 and HBc/Δ145–149), showed strongly increased (by ca. 5- to 7- fold) B701 signal relative to the WT HBc after normalization of the total HBc signal (as detected by mAb T2221) (Fig 7, lanes 4–6), indicative of enhanced CTD phosphorylation at the B701 epitope. The linker substitution mutant, HBc-LN, also showed a similar effect on CTD phosphorylation to the linker deletion mutants, albeit to a lesser degree (by ca. 3-fold) (Fig 7, lane 8). On the other hand, the 25–7 signal for the complete linker deletion (HBc/Δ141–149), the C-terminal partial linker deletion (HBc/Δ145–149), and the LN substitution mutants was modestly (by ca. 2-fold) increased relative to the WT HBc (Fig 7, lanes 4, 5, 8), suggesting the 25–7 epitope was less phosphorylated in these mutants as compared to the WT HBc.

## Discussion

**Fig 8:**
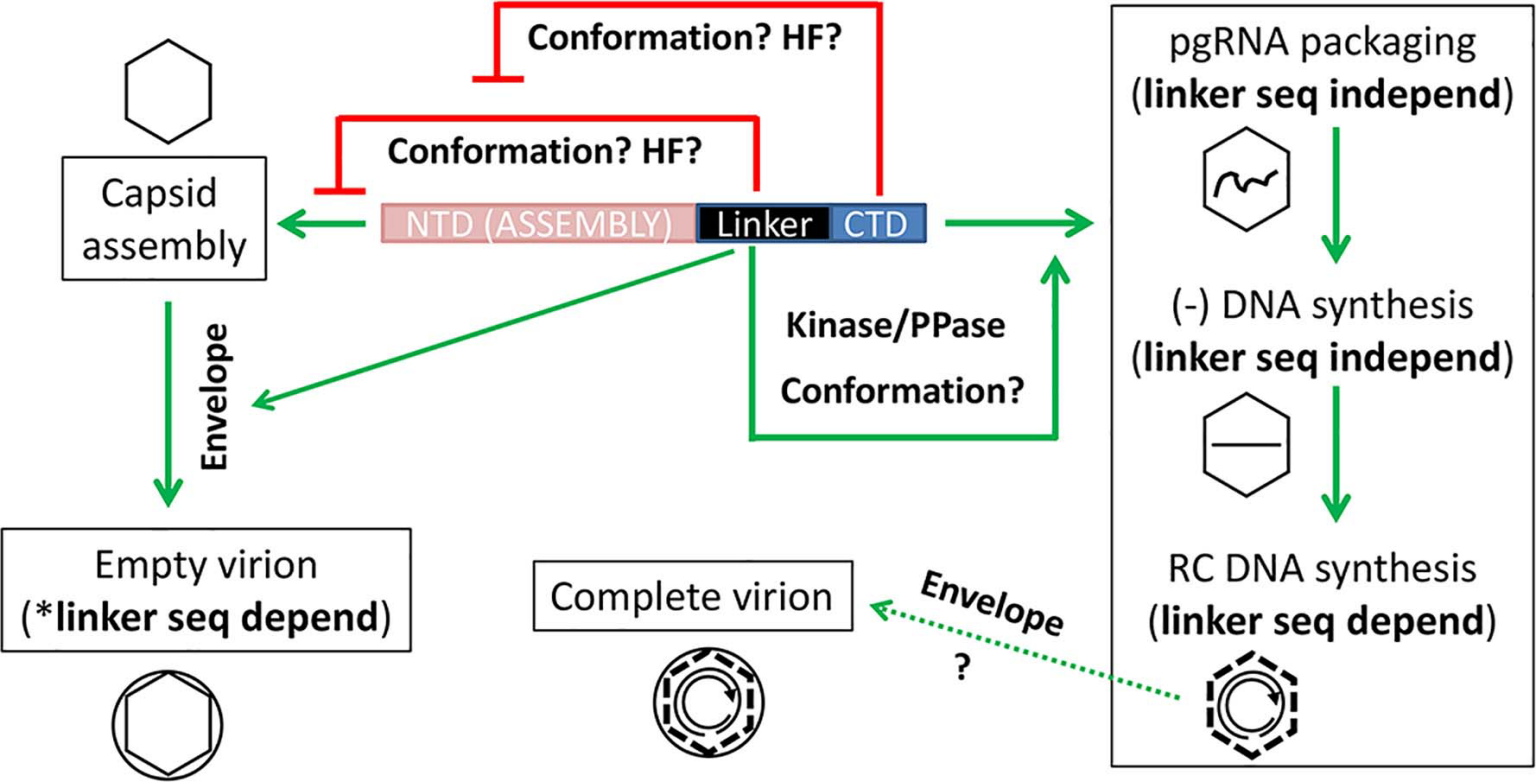
Working model for HBc linker functions at multiple stages of HBV replication. The linker is proposed to exert its effects on capsid assembly via the NTD, its effects on pgRNA packaging and DNA synthesis via the CTD, and its effects on virion secretion via interaction with the envelope proteins. Capsids are denoted by the hexagons, the viral envelope by the circles, pgRNA by the wavy line, SS DNA by the straight line, and RC DNA by the broken double concentric circle with the arrow pointing to the 3’ end of the plus strand. The broken line of the RC DNA-containing (i.e., mature) NCs denotes the fact that mature NCs are destabilized. The red lines denote inhibitory or negative effects; green lines, stimulatory or positive. The putative mediators of the negative and positive effects are also indicated, including conformation–NTD or CTD conformations involved in assembly or the other indicated aspects of HBc functions; putative host factors (HF) regulating capsid assembly; host protein kinase or protein phosphatase (PPase) that control CTD state of phosphorylation. The putative interactions between the HBc linker and the envelope proteins in virion secretion are also denoted with the green lines. seq, sequence; (in)depend, (in)dependent; *, secretion of empty virions being the most sequence-specific function of the linker. See text for details.

We have demonstrated here that mutations of the HBc linker affected multiple steps in HBV replication, including modulation of capsid assembly, pgRNA packaging, DNA synthesis, and virion secretion, implicating a critical role for the linker in multiple stages of HBV replication (Fig 1). The mechanisms of action for these linker functions remain to be elucidated. As the nine-residue long linker peptide is not known to have any enzymatic function or biochemical activity (such as nucleic acid binding), we consider it plausible that the effects of the linker on the HBc functions in capsid assembly, pgRNA packaging, and reverse transcription, are exerted through its effects on the NTD or CTD (Fig 8). This is supported by our findings that the linker affected NTD assembly and CTD state of phosphorylation. On the other hand, the linker may function in a more direct manner (independent of its effects on the NTD or CTD) to facilitate virion secretion by interacting with the viral envelope proteins (Fig 8).

As introduced earlier, in the absence of CTD, the linker is not required for capsid assembly in bacteria or under in vitro assembly conditions with high HBc and/or salt concentration [24,25]. However, deletion of the linker, thus fusing the CTD to NTD, or substitution of the linker sequence, interfered with NTD assembly in bacteria (Fig 1) [24]. In sharp contrast, we have shown here that in human hepatoma cells, the linker interfered with NTD assembly if the CTD was absent, but in the presence of the CTD, linker deletions or substitutions did not interfere with capsid assembly. Consistent with the inhibitory effect of the linker on assembly by NTD reported here, a recent study also found that HBc149 failed to accumulate in a mouse hepatocyte cell line but HBc144 did (similar to HBc143 here) [38]. It thus appears that the NTD alone is sufficient, at least to a limited extent, for capsid assembly in human cells, but the presence of the linker, in the absence of the CTD, interferes with NTD assembly specifically in human cells but not in bacteria. Furthermore, we have shown here that in the absence of CTD, deletion of the linker sequence 141–144 (HBc149/Δ141–144) was less effective in restoring capsid assembly, compared to deletion of the entire linker (in HBc140) or 144–149 (in HBc143). This suggests that N- and C-terminal sequences of the linker are not equivalent in modulating capsid assembly and the C-terminal part of the linker (144–149) may have a more detrimental effect on NTD assembly than the N-terminal part of the linker (141–144) when the CTD is absent.

How the linker may influence capsid assembly, in a host cell- and CTD-dependent manner, is one of the intriguing questions brought up by our studies here that warrants further studies. The linker may interfere with NTD assembly in human cells, in the absence of CTD, by affecting the conformation of NTD, or by interacting with a host factor(s) to inhibit assembly (Fig 8). When expressed in bacteria, the high protein concentration achieved may somehow overcome the inhibitory effects of the linker on NTD assembly, or host cell-specific factors may alleviate the linker effect. As HBc149-4R, in contrast to HBc149, assembled efficiently in human cells, a role for electrostatic interactions between the highly basic CTD and a negatively charged ligand (e.g., RNA, or acidic residues in the HBc NTD) can be implicated in alleviating the inhibitory effect of the linker on NTD assembly in human cells by the CTD. Moreover, capsid stability, instead of or in addition to assembly, could be affected by the linker, as suggested by the apparent disruption of the HBc/Δ141–149 (with the complete linker deletion, fusing the CTD directly to the NTD) capsid once it was released extracellularly.

The linker, and its specific sequences, are important for capsid assembly in bacteria when both the NTD and CTD are present (i.e., in the context of the full-length HBc) [24], but not in human cells as we have shown here. Other than the differences in HBc subunit concentration and salt/pH conditions, phosphorylation of the HBc CTD, which occurs in human cells but not in bacteria and is furthermore modulated by the linker as we have shown here, is known to modulate capsid assembly [35]. This host cell-dependent and linker-modulated CTD phosphorylation (Fig 8) may be part of the reason why deleting the linker or substituting its sequences interferes with capsid assembly in bacteria but not in human cells. In addition, CTD is known to interact with host factors in mammalian cells, such as I2PP2A and B23 [45], and SRPK [46], which may also contribute to the host cell-dependent effects of the linker mutations. Indeed, we have shown previously that the binding of I2PP2A and B23 to the CTD is modulated by the linker in the case of the duck hepatitis B virus core protein [45], which is thought to be much longer than the HBc linker and located between position 186–230 [47].

It remains possible that deletion of the CTD impaired the production and/or stability of the mutant protein in human cells (but not in bacteria), accounting for the very low expression level of HBc149 in hepatoma cells. However, we believe that the lower expression level of this mutant was mostly due to its defect in efficient assembly in mammalian cells (and consequently, more rapid degradation). First, we have shown recently that this same mutant is expressed at levels equal to or higher than the WT HBc in a mammalian cell extract, the rabbit reticulocyte lysate in vitro translation system; yet, it still fails to assemble, unlike the WT HBc in the same system that assembles efficiently [35]. Second, HBc149 expression and assembly in human cells can both be rescued by co-expression of the WT HBc [35].

Deletion of the entire linker severely impaired pgRNA packaging, and partial deletion of sequences from 145–149 had a more deleterious effect than that of 141–144, suggesting that the linker sequences from 145–149 had a more important role than 141–144 in pgRNA packaging in the presence of the CTD, similar to the non-equivalent role of the two parts of the linker on capsid assembly in the absence of the CTD. On the other hand, none of the linker residues individually was absolutely required for pgRNA packaging as they could be substituted without affecting pgRNA packaging. One potential mechanism for the linker to modulate pgRNA packaging may be via its influence on CTD phosphorylation (Fig 8), which we could demonstrate here. As we proposed recently [35], hyper- or hypo-phosphorylation of HBc CTD can both impair specific pgRNA packaging, by decreasing overall RNA (including the specific pgRNA) binding affinity or failing to block non-specific RNA binding, respectively. Details of the effects of the linker on CTD phosphorylation, in a phosphorylation site- and maturation stage-specific manner will require comprehensive studies in the future. How the linker may affect CTD phosphorylation state also remains to be elucidated. One possibility is that the linker modulates CTD conformation, which in turn affects the accessibility of the CTD phosphorylation sites to host kinases and/or phosphatases. Alternatively, the linker may affect the recruitment of these CTD-modifying host enzymes, either directly by serving as binding sites for these factors, or through an indirect means (Fig 8). Additional effects of the linker, beyond affecting CTD phosphorylation, including its influence on NTD assembly, may also play a role in modulating pgRNA packaging.

Deletion of the entire linker, or partial deletion of the linker sequences from 145–149 abolished viral reverse transcription, whereas deletion of 141–144 had only a modest effect. This result again suggests that the linker sequences from 145–149 had a more important role than 141–144 in reverse transcription, as in pgRNA packaging. However, since the linker is nine-residues long, it was impossible to construct a deletion mutant removing precisely half of the linker (i.e., 4.5 residues). So, it remains possible that HBc/Δ141–144 was more effective than HBc/Δ145–149 in making immature DNA (and packaging pgRNA) simply because it is one residue longer than HBc/Δ145–149. On the other hand, as with pgRNA packaging, it is clear that none of the linker residues individually was required for SS DNA synthesis as they could be substituted with little effect on SS DNA levels. Furthermore, our results here have shown that a linker that is five (instead of nine as in the case of the WT)-residues long is still capable of supporting pgRNA packaging and SS DNA synthesis, at least partially. This is consistent with the observation that the linker is disordered in recombinant capsids assembled in bacteria from HBc149 (i.e., missing the entire CTD) [9] and the notion that the linker may form a flexible, mobile array on the inner surface [24] of the maturing NC to facilitate this stage of viral DNA synthesis. On the other hand, RC DNA synthesis was clearly impaired by two of the three linker substitutions as well as the partial deletion from 141–144, which had little effect on SS DNA synthesis. The remaining linker substitution that was competent for RC DNA synthesis is a very conservative one with almost identical sequence and predicted structure to the WT linker. Thus, for RC DNA production, the linker did not merely function as a flexible spacer but played a specific role. How the linker might facilitate RC DNA synthesis in a sequence-dependent manner is not yet known. As the CTD state of phosphorylation is known to be important for RC DNA synthesis [17,19,20], and the linker sequences could affect CTD phosphorylation, the specific linker sequences could modulate RC DNA synthesis through their effect on CTD phosphorylation (Fig 8), as proposed above for their effects on pgRNA packaging. In addition, the linker may be involved in the conformational changes of the maturing NC that accompany, and may be required for, RC DNA synthesis [48]. In addition, the linker itself may undergo conformational changes, in a sequence-dependent manner, during the viral replication cycle that are modulated by the NTD or CTD. We note also that although no exogenous nuclease digestion was used during viral DNA extraction, our results here can’t exclude the possibility that in those mutants where no RC DNA was detectable, some RC DNA might actually have been made but degraded as soon as it was made in the cell.

Perhaps the most intriguing result we have obtained here regarding the linker functions is its essential role in the secretion of empty virions. Those linker deletion and substitution mutants that impaired RC DNA synthesis were also defective in the secretion of complete (RC DNA-containing) virions. The conservative linker substitution (LC) that remained competent for RC DNA synthesis was also capable of secreting complete virions. This is expected as RC DNA synthesis is required for complete virion formation. A specific effect of these mutants on the secretion of complete virions, however, could not be ascertained from these results (Fig 8). On the other hand, it is clear from our results here that the specific linker sequence is critical for empty virion secretion. All linker mutations, either complete or partial deletions or substitutions, impaired secretion of empty virions. Even the conservative linker substitution (LC), which was fully competent in all other aspects of the viral life cycle tested here including the secretion of complete virions, showed a severe defect (though not as severe as the other linker substitution or deletion mutants) in the secretion of empty virions. The LC linker substitution increased the ratio of complete to empty virions, by ca. 10-fold from ca. 1% to 10% [32], by decreasing the secretion of empty virions without affecting that of complete virions. Thus, we have not only uncovered an essential role of the linker in the secretion of empty virions, but also revealed that the requirements for the secretion of complete vs. empty virions can be separated genetically.

The efficient secretion of HBc149-4R capsids in empty virions further suggests that the linker is not only necessary but may be sufficient to support empty virion formation, although it remains formally possible that both the linker and several R residues from the CTD are required for the secretion of empty virions. As the beginning of the HBc CTD has the sequence _150_RRRGR_154_…, it may be argued that HBc149-4R actually retains a severely truncated “CTD,” i.e., the first three (or five without G153) residues of the CTD. Thus, further studies will be needed to clarify the contribution of the CTD, if any, in the secretion of empty HBV virions. It was recently reported that HBc147 capsids (missing the entire CTD and two C-terminal residues of the linker) failed to be secreted in empty virions [36]. Whereas the authors hypothesized that their result implicated a critical role for the CTD in empty virion secretion, our findings here suggest an alternative interpretation of the same result, i.e., the last two residues of the linker (148 and 149) plays a critical role in supporting empty virions secretion. The CTD state of phosphorylation, which appeared to be affected by the linker mutations, is unlikely to account for the effect of the linker mutations on virion secretion as CTD phosphorylation state, per se, does not play a critical role in virion formation [18]. These results, combined, suggest the intriguing possibility that linker residues interact, directly, with the envelope proteins during virion formation (Fig 8). Whereas the linker is generally thought to be located inside the capsid [24,49] and thus unlikely to interact with the envelope proteins on the capsid surface, it may nevertheless be exposed, at least transiently, on the capsid surface. Some evidence in support of an exterior localization of the linker has indeed been presented; for example, epitopes attached to the linker are accessible to antibody binding in empty HBV capsids [50,51]. Additionally or alternatively, the linker sequence may be involved, perhaps via interactions with host factors, in trafficking of the capsids to the site of budding for their envelopment.

Future studies, including high-resolution structural analysis, will be required to further elucidate the mechanisms of action of the linker functions uncovered here and to determine any structural changes in the capsid, at the various stages of viral replication, that may be modulated by the linker. For example, whereas the linker is known to affect the dichotomy of T = 4 or T = 3 capsids in bacteria, whether this is also the case in human cells remains to be determined. Furthermore, it remains unknown if both size classes of capsids are competent in pgRNA packaging or reverse transcription. On the other hand, both T = 3 and T = 4 capsids are found in extracellular virions [52], indicating that they are both competent for virion formation and so the capsid size is unlikely to be a determinant of virion formation. As uncovered here, the critical roles that the HBc linker plays at multiple stages of HBV replication, which have been thought to involve only the HBc NTD and/or CTD, emphasize the close and dynamic interactions among all three regions of HBc that together carry out the multiple essential functions of HBc in viral replication. As conformational changes are likely to be associated with NC maturation and envelopment [6,30,32,48,53], further structural studies of the HBc linker mutants that affect various stages of viral replication should provide important insights into the effects of the linker on the conformations of the HBc NTD, CTD, and the NC as a whole and how the conformational effects translate to functional effects on NC maturation and envelopment.

The multiple roles of the HBc linker in HBV replication that we uncovered here provide an explanation for the high degree of sequence conservation in this region of HBc. In addition, as the same DNA sequence coding for the HBc linker also codes for the very N-terminal part (residues 5–14) of the viral RT protein, the need to preserve polymerase sequence and functions possibly also has contributed to the conservation of the DNA sequence in this region of the HBV genome. However, as we highlighted recently [54], the N-terminal sequences of the polymerase are actually not highly conserved and mutagenesis work so far indicates that this region of the polymerase is not essential for any known functions of the polymerase although it does contribute, to some degree, to the polymerase functions in pgRNA packaging and protein-primed initiation of reverse transcription. Thus, it is likely that the preservation of the HBc linker sequence and functions has played a more important role in the DNA sequence conservation of this region of the viral genome. On the other hand, some variations of the linker sequence have been observed [23]. In light of our findings here, future studies to examine the functional effects of the naturally occurring linker variations are warranted.

HBc has emerged recently as the primary target, after the HBV RT protein, for developing effective antiviral strategies to clear HBV infection. Almost all agents in development so far are targeted to the NTD [5,55,56]. Our results here indicate that sequences outside the NTD, including the CTD as well as the linker, could represent important targets for HBc-directed antiviral development. In fact, a small molecule compound has been reported recently that inhibits HBc assembly and functions in a manner that is dependent on sequences in the CTD [57]. Similarly, it may be possible to identify compounds that target the conserved HBc linker region to inhibit multiple steps of HBV replication. Our discovery here of the multiple critical functions of the HBc linker in HBV replication also has broad implications. Thus, linkers connecting protein domains are common occurrences including those in other viral capsid proteins [58]. For the human immunodeficiency virus type 1 (HIV-1), the linker in its capsid protein has been shown to regulate capsid stability and reverse transcription [59].

## Materials and methods

### Plasmids

pCI-HBc and -HBc149 expressing the full-length and CTD-deleted HBc have been described before [35]. pCI-HBc149-4R is identical to pCI-HBc149, except four R residues are added after HBc position 149 (Fig 1). pCI-HBc140, -HBc143, -HBc149/Δ141–144, -HBc/Δ141–149, -HBc/Δ141–144, -HBc/Δ145–149 were derived from pCI-HBc through PCR-mediated mutagenesis for the expression of CTD and/or linker deletion mutants (Fig 1). Three linker substitution mutants of HBc were also constructed via PCR mutagenesis. The C-terminal seven residues of the linker were randomized in sequence in the mutant LR, or replaced with the seven N-terminal residues of HBc in LN as described before [24]. In the third substitution mutant, LC, the entire linker was replaced with a nine-residue segment from a cellular protein (cellobiose dehydrogenase) similar in sequence and predicted structure to the linker [24] (Fig 1). pSV-HBV1.5/C^-^ expresses a HBc-defective HBV genome [30], which is capable of supporting viral replication upon complementation with HBc. pCMV-HBV expresses the HBV pgRNA from the heterologous cytomegalovirus (CMV) immediate early promoter and the HBV surface mRNAs from the endogenous HBV promoter, leading the production of all viral RNAs and proteins required for replication and virion secretion [60,61].

### Antibodies

A mouse monoclonal antibody (mAb), clone T2221, against the HBc NTD [39] was purchased from Tokyo Future Style (Cat no. 2AHC24). The mAb 10E11 against HBc NTD (residues 2–10) [40] was purchased from Abcam (Cat no. ab8639). The mAb, anti-WHc, specific for the WHc NTD (likely the first 8 residues), is cross-reactive with HBc due to the identity of the very N-terminal HBc and WHc sequences, as reported before [32,41]. The HBc CTD-specific mAbs, 25–7 and B701, have been described recently [18,35]. The rabbit polyclonal antibody against HBc were purchased from Dako. The rabbit anti-HBs polyclonal antibody was purchased from Virostat [18]. The anti-preS2 mAb (Arigo Biolaboratories) detect the preS2 region that is shared by both the L and M (but absent from the S) HBV envelope proteins.

### Transient transfection and analysis of core protein expression and viral DNA synthesis

HBc expression constructs and/or HBV genomic constructs were transfected into the human hepatoma cell line HepG2 or Huh7 cells (kindly provided by Christoph Seeger, Fox Chase Cancer Center) as previously described [42,62,63]. Briefly, HepG2 cells in 60-mm dishes were transfected with 4 μg of plasmid using FuGENE6 (Roche). Huh7 cells seeded in 60-mm dishes were transfected with 10 μg of plasmid using CalPhos Mammalian Transfection Kit (Clontech). Cells and culture supernatant were harvested on day 7 post-transfection. Cells were lysed with NP40 and HBc proteins in the cytoplasmic lysate were resolved by sodium dodecyl sulfate-polyacrylamide gel electrophoresis (SDS-PAGE), transferred to polyvinylidene difluoride (PVDF) membrane, and detected by the indicated antibodies as described previously [19,32]. Core DNA from NCs was isolated from the cytoplasmic lysate without nuclease digestion and analyzed by Southern blot analysis as described previously [42]. A genome-length, ^32^P-labeled HBV DNA probe was used to detect the viral DNA replicative intermediates by Southern blot analysis.

### Native agarose gel electrophoresis for analyzing NC assembly and virion secretion

Native agarose gel electrophoresis of intact NCs from the cytoplasmic lysate, or extracellular viral particles obtained after DNase I digestion of polyethylene glycol (PEG) precipitated cell culture supernatant [32] were carried out by using previously reported procedures [18,19,31,32]. Briefly, following transfer to nitrocellulose membrane, viral DNA associated with the particles was detected using a full-length HBV DNA probe, or pgRNA packaged into NCs by using a minus-sense riboprobe. The same membrane was subsequently probed with the indicated HBc or surface specific antibody to detect HBc or surface proteins. The signals from the ^32^P-labeled RNA probe were quantified using a phosphor imaging system (GE Healthcare). The chemiluminescent signals representing the capsid protein were quantified using the ChemiDoc MP system and BioLab software, as previously described [64]. Densitometry using appropriately exposed films was also used in some cases to quantify the RNA and protein signals. All quantifications were repeated with at least three separate transfection experiments.

### *In vitro* translation in rabbit reticulocyte lysate (RRL)

A TnT-coupled rabbit reticulocyte lysate (RRL) *in vitro* translation system (Promega) was used to express the WT HBc or linker deletion/substitution mutants, as described previously [35]. *In vitro*-translated proteins were analyzed by SDS-PAGE and western blot using the indicated anti-HBc antibodies.

## Supporting information

S1 Fig Levels of HBsAg secretion from the different HBV constructs in transfected hepatoma cells. Huh7 cells were transfected with two different HBV genomic constructs as indicated. Seven days later, the culture supernatant was collected. Concentrated culture supernatant was analyzed for secretion of the viral envelope proteins (HBs). Following agarose gel electrophoresis and transfer to nitrocellulose membrane, the envelope proteins were detected by using the anti-HBs antibody.
(TIF)

S2 Fig Comparison of mAb T2221 and two other HBc NTD mAbs for detection of HBc proteins. Cytoplasmic lysate from HepG2 cells transfected with the expression construct for either the WT HBc (lanes 1, 4, 7), HBc140 (lanes 2, 5, 8), or HBc143 (lanes 3, 6, 9) were resolved by SDS-PAGE. Following transfer to PVDF membrane, HBc proteins were detected by using the indicated mAbs, 10E11 (lanes 1–3), Anti-WHc (lanes 4–6), and T2221 (lanes 7–9). C, HBc; C^T^, truncated HBc (HBc140, HBc143).
(TIF)
