# Supplementary figures and images for: Multiple roles of core protein linker in hepatitis B virus replication
Source: PLoS Pathog. 2018 May 21;14(5):e1007085. doi: 10.1371/journal.ppat.1007085 (PMC5983865; doi:10.1371/journal.ppat.1007085)

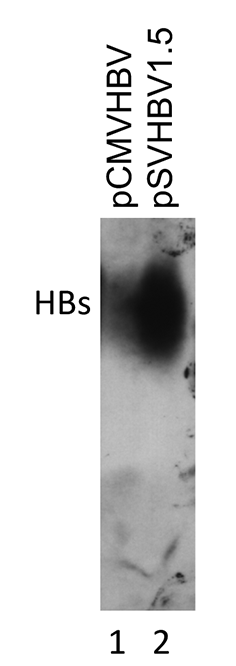

Supplement: S1 Fig — Huh7 cells were transfected with two different HBV genomic constructs as indicated. Seven days later, the culture supernatant was collected. Concentrated culture supernatant was analyzed for secretion of the viral envelope proteins (HBs). Following agarose gel electrophoresis and transfer to nitrocellulose membrane, the envelope proteins were detected by using the anti-HBs antibody. (TIF) [file ppat.1007085.s001.tif]

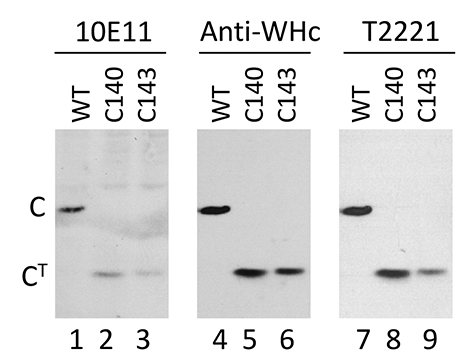

Supplement: S2 Fig — Cytoplasmic lysate from HepG2 cells transfected with the expression construct for either the WT HBc (lanes 1, 4, 7), HBc140 (lanes 2, 5, 8), or HBc143 (lanes 3, 6, 9) were resolved by SDS-PAGE. Following transfer to PVDF membrane, HBc proteins were detected by using the indicated mAbs, 10E11 (lanes 1–3), Anti-WHc (lanes 4–6), and T2221 (lanes 7–9). C, HBc; CT, truncated HBc (HBc140, HBc143). (TIF) [file ppat.1007085.s002.tif]
